# Attenuation of autophagy impacts on muscle fibre development, starvation induced stress and fibre regeneration following acute injury

**DOI:** 10.1038/s41598-018-27429-7

**Published:** 2018-06-13

**Authors:** Andrea Paolini, Saleh Omairi, Robert Mitchell, Danielle Vaughan, Antonios Matsakas, Sakthivel Vaiyapuri, Thomas Ricketts, David C. Rubinsztein, Ketan Patel

**Affiliations:** 1 School of Biological Sciences, University of Reading, Reading, UK; 2 Molecular Physiology Laboratory, Centre for Atherothrombotic & Metabolic Disease, Hull York Medical School, Hull, UK; 3 School of Pharmacy, University of Reading, Reading, UK; 4 Cambridge Institute for Medical Research, Department of Medical Genetics, University of Cambridge, Cambridge, UK; 5 UK Dementia Research Institute, Cambridge Biomedical Campus, Cambridge, UK

**Keywords:** Autophagy, Proteolysis

## Abstract

Autophagy has been implicated as a major factor in the development of a number of diseases of skeletal muscle. However, its role in skeletal muscle homeostasis is still evolving. We examined skeletal muscle architecture in a mouse model, *Atg16L1*, where autophagy is attenuated but importantly still present. We show that muscle fibres from *Atg16L1 mice* were smaller than wild-type counterparts, proving a role for this process in the growth of these cells. We show that mild attenuation of autophagy results in accelerated muscle loss during the initial phase of acute starvation. Furthermore, we show that regeneration of skeletal muscle following cardiotoxin (CTX) mediated injury is slower in the *Atg16L1* mouse compared to wild-type. Lastly, we show that autophagy controls the integrity of the sarcolemma. Attenuated autophagy makes muscle fibres more susceptible to infiltration by circulating immunoglobulins following muscle injury with CTX. These fibres internalise dystrophin and nNOS. Importantly these fibres are able to restore dystrophin and nNOS localisation and do not die. In conclusion, these studies shed new light into the ability of skeletal muscle fibres to cope with injury and establish a link between the fine-tuning of autophagy and skeletal muscle regeneration.

## Introduction

Maintenance of cellular homeostasis requires robust mechanisms to remove misfolded proteins, and in the case of eukaryotes, aberrant organelles. Both bacteria and eukaryotes degrade proteins in a subcellular structure called the proteasome^1^. In bacteria, a signal encoded in the primary protein sequence identifies polypeptides destined for proteasome degradation. In contrast, eukaryotes mark proteasome substrates through poly-ubiquitination^2^. Unlike prokaryotes, eukaryotes also contain membrane bound organelles which often malfunction and need to be eliminated. Removal of bulk cytoplasmic content and organelles in eukaryotes is mediated by one of two processes called microautophagy or macroautophagy. Microautophagy is the direct engulfment of organelles and cytoplasm by the lysosome. In contrast macroautophagy, hereafter referred to as autophagy, involves the formation of a double membrane structure around the degradation target, called the autophagosome, which subsequently fuses with the lysosome where the final process of breakdown occurs^3^.

Studies on autophagy stretch back to the 1950’s with the pioneering, mainly descriptive work of de Duve^4^. However, the molecular players in this process were not characterised not until the later part of the 20^th^ century and the beginning of the 21^st^ often using yeast as a model organism. A large number of autophagy related genes (ATG) have been identified (to date more than 30) in this organism that participate in this process^5^. Autophagy starts with the formation of a cup-like membrane structure called the phagopore. Autophagy can be divided into five key stages: (1) The phagopore formation or nucleation, a process that requires VSP34 in conjunction with many proteins including Beclin 1 to produce PI(3)P. (2) The conjugation of multimeric Atg5-Atg12-Atg16L complex to the extending phagopore, a process thought to induce curvature of the growing phagopore. (3) Processing and conjugation of microtubule-associated protein light chain 3 (LC3) into the phagopore. Maturation of LC3 requires it be lipidated through its conjugation with phosphatidylethanoalamine (PE). LC3 is proposed to be involved in the selection of proteins destined for autophagy^6^. (4) Capture of cytosol and/or organelle for degradation marked by the formation of the autophagosome (5) Fusion of autophagosome with the lysosome.

Given that autophagy is a major mechanism that controls cellular content, it is hardly surprising that its deregulation plays a significant part in the development of human diseases. Mutations in key autophagy genes have been identified in a spectrum of ailments ranging from cancer, neurodegenerative diseases to diseases of muscle (reviewed by Jiang and Mizushima^7^). Many human diseases have been modelled in mice through autophagy gene deletion or over ablation studies. Although these studies have been extremely instructive in understanding the role of autophagy during disease progression, they, by their robust nature, give relatively limited insights into the impact of this mechanism of tissue homeostasis. To achieve the later, a number of investigators have developed models in which autophagy is mildly increased or reduced. One such model which has proven the value of such an approach is the hypomorphic *Atg16L1* mouse. Atg16L is a key component of autophagy. It forms a complex with Atg12 and Atg5 and has been shown to specify the site of LC3 lipidation of membrane biogenesis^8^. Cadwell and colleagues developed the *Atg16L* hypomorph mouse model called *Atg16L1* in which the expression of the gene was diminished, but importantly not absent^9^. The *Atg16L* hypomorph mouse has decreased levels in autophagy in all tissues examined to date. This mouse model was used to show that autophagy plays a role in the development of Crohn’s disease by controlling the activity of intestinal Paneth cells^9^. This mouse model has been extensively used to study the role of autophagy in other tissues and recently been shown to control stem cells developing in the brain, gut and bone^10^. The value of a hypomorph approach was exemplified in the later study, which showed that when autophagy was reduced, the outcome manifests in stem cells being maintained in an undifferentiated state. In contrast when key autophagy genes have been ablated this results in apoptosis of the neural stem cells^11^.

A large number of studies have investigated the role of autophagy in skeletal muscle development and its function. Most have relied on gene knock-out or over-expression strategies. These have shown that over-active or an absence of autophagy has a major impact on muscle mass and its ability to generate force^12,13^. However very few have examined the physiological role played by autophagy in skeletal muscle. Here we have carried out a detailed investigation of the skeletal muscle of the *Atg16L1* mouse.

We show that autophagy plays a role in the hypertrophy of muscle fibres, allowing them to attain normal size. Additionally, we show that autophagy plays a significant role in coping with starvation induced stress and during the regeneration process following acute injury. Furthermore, we show a novel role for autophagy in maintain sarcolemma integrity.

## Results

### Autophagy and muscle fibre growth

**Figure 1:**
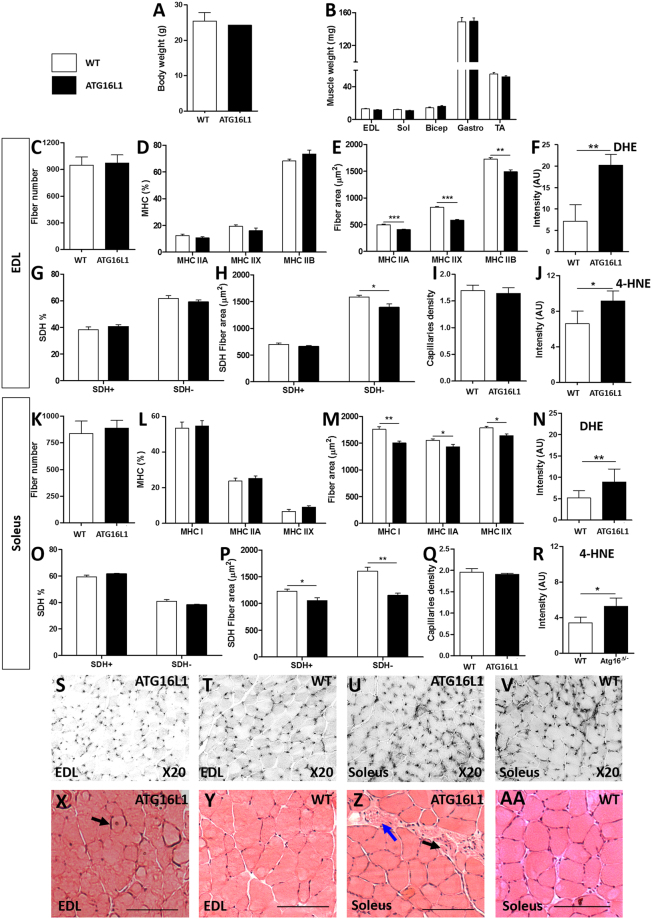
Characterisation of *Atg16L1* skeletal muscle from male mice. (**A**) Body weight. (**B**) Weight of hind limb muscles. (**C**) Muscle fibre count at the mid-belly of the EDL. (**D**) MHC profile of EDL muscle. (**E**) MHC fibre size of EDL muscle. (**F**) Quantification of ROS levels using DHE in the EDL muscle. (**G**) Profiling the proportion of SHD positive fibres in the EDL. (**H**) SDH fibre size profiling of the EDL. (**I**) EDL capillary density. (**J**) Quantification of lipid peroxidation using 4-HNE in the EDL. (**K**) Muscle fibre count at the mid-belly of the soleus. (**L**) MHC profile of soleus. (**M**) MHC fibre size of the soleus. (**N**) Quantification of ROS levels using DHE in the soleus. (**O**) Profiling the proportion of SHD positive fibres in the soleus. (**P**) SDH fibre size profiling of the soleus. (**Q**) Soleus capillary density. (**R**) Quantification of lipid peroxidation using 4-HNE in the soleus. (**S**,**T**) CD31 staining to identify capillaries in EDL muscle. (**U**,**V**) CD31 staining to identify capillaries in the soleus. (**X**,**Y**) H and E stain of EDL muscle. (**Z**-AA) H and E stain of soleus muscle. Centrally located nuclei indicated by black arrow and necrosing muscle fibre highlighted with the blue arrow in ATG16L1 muscle. Scale bar represents 100 µm. n = 4–6 8-week-old-male for each cohort. *p < 0.05 and **p < 0.01. Statistical analysis between two groups performed by two-tailed Student’s t test for independent variables.

We first established the skeletal muscle phenotype of the *Atg16L1* mouse. *Atg16L1* mice were viable and were born at expected Mendelian ratios. In agreement with published works, the adult male *Atg16L1* mice in our study weighed the same as wild-types (Fig. 1A)^9^. Examination of the weights of five hind limb muscles from *Atg16L1 mice* revealed that most were lighter than their wild-type counterparts without reaching statistical significance (Fig. 1B). We thereafter carried out a detailed examination of a relatively fast contracting, glycolytic muscle, the Extensor Digitorum Longus muscle (EDL) and the soleus muscle a slow contracting oxidative muscle to reveal potentially subtle muscle phenotype as a consequent of reducing ATG16 activity. There was no significant change in either fibre number or the proportion of fibres expressing the major Myosin Heavy Chain isoforms (MHC) in the EDL and Soleus (Fig. 1C,D,K,L). However morphometric analysis revealed a small but significant decrease in the size of all MHC fibre types in both muscles (Fig. 1E,M). Profiling of muscle fibres based on oxidative metabolic activity, on the basis of the proportion of fibres containing high levels of Succinate Dehydrogenase (SDH) revealed no differences between that of *Atg16L1*and wild-type. The fibre size decrease revealed through MHC profiling was also evident when plotted against SDH activity (Fig. 1H,P). The numbers of capillaries associated with each muscle fibre (capillary density) was not affected by the *Atg16L1* mutation in either muscle (Fig. 1I,Q,S–V). Lastly, we assessed oxidative stress in the *Atg16L1*, since muscle relies on high levels of metabolic activity which generates reactive oxygen species (ROS). Firstly we assessed the levels of ROS using Dihydroethidium (DHE)^14^. We found that DHE levels were elevated in the muscle from *Atg16L1* (Fig. 1F,N). Thereafter we quantified the levels of lipid peroxidation which is caused by ROS, by examining the production of 4-hydroxy-2-nonenal (4-HNE)^15^. We found higher levels of 4-HNE in the muscle from *Atg16L1* compared to wild-type (Fig. 1J,R). Examination of muscle after Haematoxylin and Eosin staining revealed the presence of numerous aberrant fibres (with centrally located nuclei, irregular shape, extremely small size) originating from *Atg16L1* mice which were absent in wild-type tissue (Fig. 1S–V). These results show that small changes in the level of autophagy results in subtle quantitative and qualitative changes in skeletal muscle biology.

### Starvation

**Figure 2:**
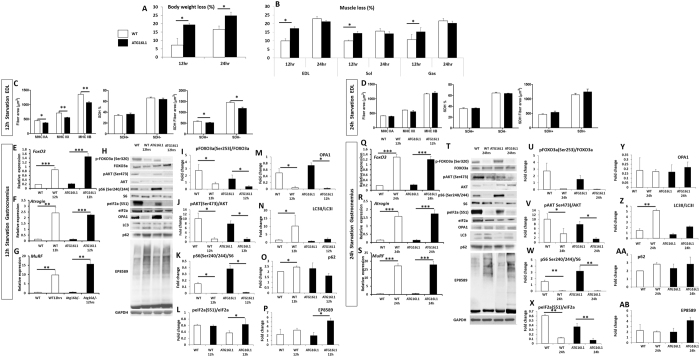
Accelerated muscle atrophy in *Atg16L1* mice induced by starvation. (**A**) Body mass decrease at 12 and 24 h after total food withdrawal. Expressed as a percentage lost compared to normally fed mice. (**B**) Muscle loss (percent of normal) following total food withdrawal. (**C**) Morphometric analysis of EDL fibres after 12 h of starvation and (**D**) at 24 h after food withdrawal. Changes in (**E**) *FoxO3*, (**F**) *Atrogin* and (**G**) *MuRF1* in the gastrocnemius after 12 h of starvation. (**H**) Western blot of key proteins from the gastrocnemius after 12 h of starvation. Quantification of gastrocnemius protein expression for (**I**) pFox3a (**J**) pAKT, (**K**) pS6, (**L**) peIF2a, (**M**) OPA1, (**N**) LC3II/LCI (**O**) p62, and (**P**) levels of total poly-ubiquitination using EP8589 after 12 h of starvation. Changes in (O) *FoxO3*, (P) *Atrogin* and (**Q**) *MuRF1* in the gastrocnemius after 24 h of starvation. (**T**) Western blot of key proteins from the gastrocnemius after 24 h of starvation. Quantification of gastrocnemius protein expression for (**U**) pFox3a (**V**) pAKT, (**W**) pS6, (**X**) peIF2a, (**Y**) OPA1, (**Z**) LC3II/LCI (AA) p62, and (AB) levels of total poly-ubiquitination using EP8589 after 24 h of starvation. n = 3–4 8-week-old-female for each cohort. *p < 0.05, **p < 0.01 and ***p < 0.001. Statistical analysis between two groups performed by two-tailed Student’s t test for independent variables.

Autophagy has been shown to protect against nutrient stress through its ability to recycle amino acids to maintain viability^16^. We determined the impact of mildly attenuating autophagy following total nutrient withdrawal, by starving the two cohorts for a maximum period of 24 h. Both wild-type and *Atg16L1* mutant mice lost body weight at both 12 h and 24 h of food deprivation (Fig. 2A). Importantly at both time points the impact on body weight loss was greater in *Atg16L1* than wild-type (Fig. 2A). This was also the case for individual muscle groups at 12 h but not at 24 h of starvation, when both cohorts had lost similar levels of muscle compared to fed mice (Fig. 2B). All fibre types, irrespective of MHC isoform expression or expression of SDH had undergone atrophy in both cohorts during the starvation experiment. Importantly, during the first 12 h of starvation, atrophy was more pronounced in the *Atg16L1* mutant mice than wild-type (Fig. 2C). However, by 24 h, both cohorts had similar sized fibres with respect to MHC isoform and SHD expression level classification (Fig. 2D). An almost identical effect of starvation was noted in the soleus muscle of *Atg16L1* mutant mice compared to wild-type (Supplementary Figure 1). We examined the mechanisms underpinning the accelerated muscle loss in the first 12 h of starvation in *Atg16L1* mutant mice. Gene expression showed that the expression of *Foxo3* a key regulator of catabolic process was induced to a greater level in *Atg16L1* mutant mice than wild-type at 12 h of starvation (Fig. 2E). We found that key genes encoding regulators of the proteasome degradation pathway, *Atrogin* and *MuRF* were robustly induced in both cohorts (Fig. 2F,G). Next, we investigated, at the protein level, the abundance of key regulators of muscle mass (Fig. 2H). The levels of active FoxO3a (determined by the phosphorylation status of Serine 320, a process mediated by kinases including Akt^17^) was increased by starvation in both cohorts (Fig. 2J). The level of activated AKT (Serine 473) as well as a downstream target (S6- Serines 240 and 244) were down-regulated by starvation at 12 h in both cohorts (Fig. 2K,J). The level of peIF2a a measure of ribosomal assembly inactivation (Serine 51) was induced by starvation only in *Atg16L1* mutant mice (Fig. 2L). Expression of OPA1, a key molecule required to maintain mitochondrial function and subcellular localisation^18^ was greatly reduced in both cohorts by 12 h of food deprivation (Fig. 2M). However, we found that whereas there was robust induction of autophagy, assessed by quantifying lipidated levels of LC3, by 12 h of starvation in wild-type mice (Fig. 2N) there was no such outcome in *Atg16L1* mutant mice (Fig. 2N). We also found that the levels of another autophagy protein, p62, were increased in WT after 12 hours of starvation, but not in muscle of *Atg16L1* mutant mice (Fig. 2O). We also investigated the levels of poly-ubiquitination and found a significantly higher degree in the muscle of *Atg16L1* mutant mice than wild-type after 12 h of starvation (Fig. 2P).

At 24 h of starvation, we detected similar elevated levels of *FoxO3*, *Atrogin* and *MuRF* in the two cohorts (Fig. 2Q,S). Western blotting showed robust increase in the levels of activated FoxO3a in both genotypes after 24 h of starvation (Fig. 2T,U). Western blot analysis showed that protein synthesis mediated by AKT through S6 were significantly inhibited in both cohorts by starvation (Fig. 2V,W). Interestingly inhibition of ribosomal assembly was alleviated by prolonged starvation in both cohorts (Fig. 2X). Expression of OPA1 was also different at 24 h compared to 12 h. Herein the levels which were previously repressed were now normal in both cohorts (Fig. 2Y). Nevertheless, autophagy as judged by the level of LC3 lipidation was elevated in muscle of wild-type mice after 24 h of starvation but had not been induced even after this time in *Atg16L1* mutant mice (Fig. 2Z). Levels of p62 were similar in the muscle of both starved cohorts (Fig. 2AA). Levels of poly-ubiquitination were similar in both cohorts at 24 h (Fig. 2AB).

### Muscle Regeneration

Next, we assessed the effect of small changes in the level of autophagy on muscle regeneration. To that end, the Tibialis Anterior muscle (TA) was damaged using cardiotoxin (CTX), a process known to kill muscle fibres without impacting on satellite cells, and examined at two separate time points at which regeneration starts (three days post-injury) and late phase of regeneration (six days post-injury)^19^.

**Figure 3:**
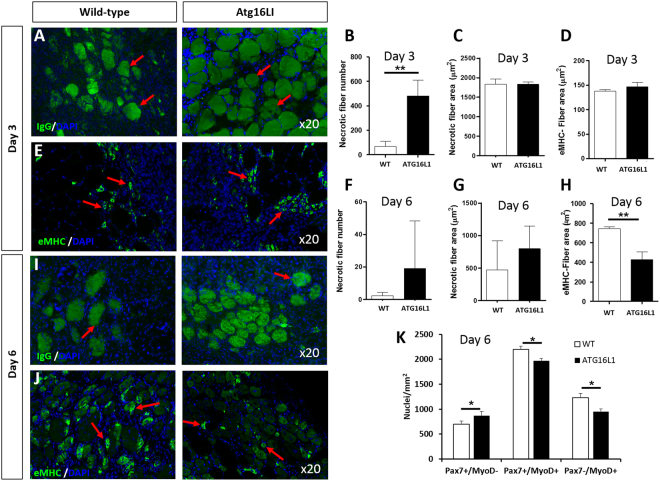
Attenuated regeneration of *Atg16L1* skeletal muscle after acute damage. (**A**) Identification of muscle fibres infiltrated by circulating Ig in wild-type and *Atg16L1* TA muscle at day 3 post injury (arrows). (**B**) Quantification of Ig infiltrated necrotic fibres, (**C**) necrotic fibre size and (**D**) size of regenerating muscle fibres 3 days after injury. (**E**) Immunocytochemical image of embryonic MHC localisation to identify regenerating muscle fibres 3 days after injury (arrows). (**F**) Quantification of Ig infiltrated necrotic fibres, (**G**) necrotic fibre size and (**H**) size of regenerating muscle fibres 6 days after injury. (**I**) Identification of muscle fibres infiltrated by circulating Ig in wild-type and *Atg16L1* TA muscle at day 6 post injury (arrows). (**J**) Immunocytochemical image of embryonic MHC localisation 6 days after injury (arrows). (**K**) Quantification of Pax7 and MyoD immunocytochemical expression in regenerating muscle at day 6 after injury. n = 3/4 8-week-old-male for each cohort. *p < 0.05, **p < 0.01. Statistical analysis between each two groups performed by two-tailed Student’s t test for independent variables.

There was a clear impact of genotype in terms of degeneration evidenced by the density of damaged fibres (assessed by the number being infiltrated by circulating Ig molecules) three days after injury. In damaged areas, there were five times more damaged muscle fibres in the TA of *Atg16L1* compared to wild-type (Fig. 3A,B). The size of damaged fibres was the same between the genotypes (Fig. 3C) as was the size of regenerating fibres (Fig. 3D,E). Six days after muscle damage, the density of damaged fibres was still higher in the *Atg16L1* albeit not reaching statistical significance (Fig. 3F,I). The size of damaged fibres had decreased to similar levels in both genotypes (Fig. 3G). However, the size of regenerating fibres was significantly smaller in the *Atg16L1* compared to wild-type (Fig. 3H,J). We profiled the status of satellite cells and their descendants to gain an insight into the attenuated muscle regeneration displayed by *Atg16L1* mice. Profiling of muscle stem cells (Pax7^+^/MyoD^−^), muscle precursors (Pax7^+^/MyoD^+^) and committed myoblasts (Pax7^−^/MyoD^+^)^20^ revealed that *Atg16L1* satellite cells were held back from differentiating, evidenced by more cells being in the stem cell state, with a deficit in numbers in both the precursor and in the committed myoblast state (Fig. 3K).

These results show that attenuated autophagy impacts on both the myofibre and muscle stem cells; making fibres more prone to infiltration after CTX administration insult and skewing the satellite cell differentiation program towards stem cell status and away from differentiation.

### Sarcolemma fragility and autophagy

**Figure 4:**
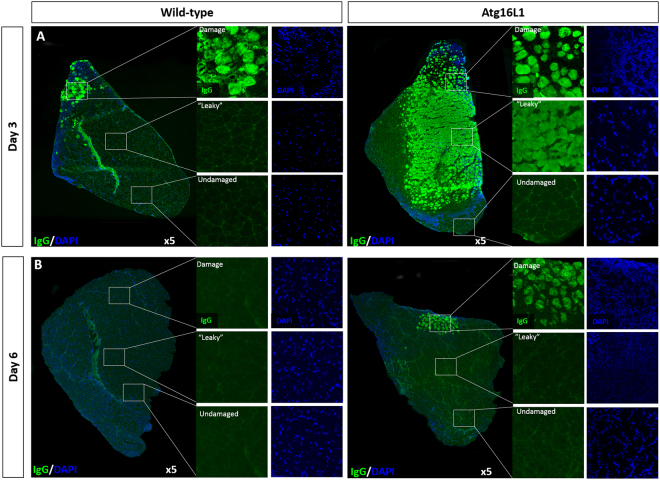
Sarcoplasmic fragility and autophagy. (**A**) Ig levels detected using immunocytochemistry three days after CTX injection. Damaged areas identified by the presence of small sized infiltrated fibres which also contain centrally located nuclei as well as huge levels of cellular infiltration. Leaky are only present at day three in muscle of *Atg16L1* TA identified by Ig infiltration but fibres in this area are regular in shape and same size as in undamaged muscle. Additionally note no centrally located nuclei or cellular infiltration (**A**) leaky area of *Atg16L1* muscle). (**B**) Infiltration of Ig in *Atg16L1* muscle resolved by day six. n = 3/4 8-week-old-male for each cohort.

During the examination of muscle following CTX injection we noted a major feature that segregated only with the *Atg16L1* mutation. We found there was extensive infiltration of circulating Igs into fibres that appeared otherwise normal, three days after CTX injection in the muscle from *Atg16L1* mice but not wild-type (Fig. 4A). We called this area ‘leaky’ which as otherwise stated appeared normal, this area had normally sized muscle fibres, the nuclei were peripherally located and there was no sign of cellular infiltration between fibres (see DAPI imaged in Fig. 4A). The leaky area covered the vast majority of the TA muscle from *Atg16L1* mice except in three areas. The first was in the damaged region which could be readily identified by the significant level of cellular infiltration between damaged fibres, with the fibres in that area also smaller than in other regions. There were an additional two regions where fibres were of normal size and lacked intra-fibre Ig infiltration and inter-fibre cellular infiltration, one at a site furthest from the point of CTX injection (labelled ‘undamaged in Fig. 4A) and one lateral to the central TA tendon (identifiable by the auto-fluorescence in the wild-type image in Fig. 4A). Importantly by day six after CTX injection, the leaky area in the *Atg16L1* muscle had been completely resolved, supported by the lack of Ig molecules inside muscle fibres (Fig. 4B).

**Figure 5:**
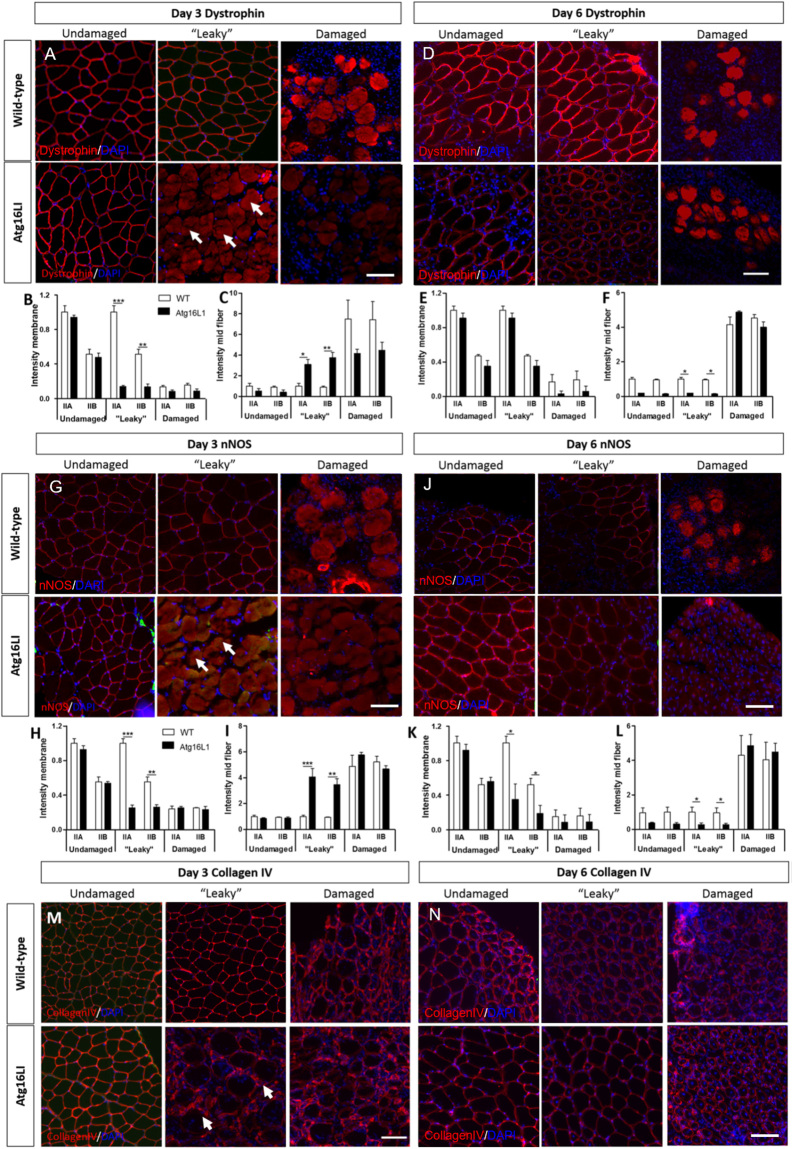
The impact of attenuated autophagy following acute muscle damage on components of the dystrophin associated glycoprotein complex. (**A**) Immunocytochemical analysis of dystrophin expression in undamaged, leaky and damaged regions at day three. Arrows show presence of dystrophin within the fibre in leaky area in Agt16L1 muscle. Quantification of dystrophin levels at the sarcolemma (**B**) and with the fibre (**C**) with relation to muscle fibre type in three regions of interest at day three. Note all quantification levels compared to a baseline of 1 of undamaged type IIA fibres. (**D**) Immunocytochemical analysis of dystrophin expression in undamaged, leaky and damaged regions at day six. Quantification of dystrophin levels at the sarcolemma (**E**) and with the fibre (**F**) with relation to muscle fibre type in three regions of interest at day six. (**G**) Immunocytochemical analysis of nNOS expression in undamaged, leaky and damaged regions at day three. Arrows highlight nNOS within the fibre in leaky area in Agt16L1 muscle. Quantification of nNOS levels at the sarcolemma (**H**) and with the fibre **(I**) with relation to muscle fibre type in three regions of interest at day three. (**J**) Immunocytochemical analysis of nNOS expression in undamaged, leaky and damaged regions at day six. Quantification of nNOS levels at the sarcolemma (**K**) and with the fibre (**L**) with relation to muscle fibre type in three regions of interest at day six. Immunocytochemical analysis of Collagen IV expression at day three (**M**) and day six (**N**) in undamaged, leaky and damaged regions in the two cohorts. Arrows in (**M**) highlight non-uniform collagen expression in Atg16L1 muscle at day three. Scale bar 100 µm. n = 3/4 8-week-old-male for each cohort. *p < 0.05, **p < 0.01 and ***p < 0.001. Statistical analysis between two groups performed by two-tailed Student’s t test for independent variables.

We investigated whether the leakiness of *Atg16L1* muscle fibres following exposure to CTX had an impact on key muscle proteins. To that end we focused on key molecules (dystrophin, nNOS and collagen IV) that protect muscle from contraction mediated damage and profiled their distribution in undamaged, leaky and damaged regions in the two cohorts^21^. Dystrophin was localised to the sarcolemma in both cohorts at equal levels (gauged by semi-quantitative immunofluorescence) in undamaged regions (Fig. 5A–F). In damaged regions of both cohorts, we found that dystrophin was present but was localised to an equal extent within the muscle fibre (Fig. 5A,C,D,F). The most striking difference at day three with regards to dystrophin localisation between the two cohorts was in the leaky region; the wild-type muscle has a pattern like that of undamaged region, whereas corresponding region of *Atg16L1* muscle showed presence of dystrophin within the muscle fibre (Fig. 5A–C). By day six, dystrophin localisation within the leaky area of *Atg16L1* muscle had reverted to the normal sarcolemma position albeit at lower levels than wild-type (Fig. 5D–F). Next, we examined the impact of leaky fibres on the localisation of nNOS at the sarcolemma, which is totally dependent on its interaction with dystrophin^22^. Both at the qualitative and quantitative levels, we found that the distribution of nNOS mirrored that of dystrophin; nNOS was localised at the muscle sarcolemma in undamaged regions in both cohorts but in the fibre in damaged regions (Fig. 5G–I). Additionally, like dystrophin, the leaky area showed intra-fibre localisation of nNOS only in the *Atg16L1* muscle at day three (Fig. 5G–I) which was resolved by day 6 (Fig. 5J). However, levels of nNOS at the sarcolemma of leaky fibres were lower in *Atg16L1* muscle than wild-type (Fig. 5K,L). Lastly we examined the localisation and levels of a collagen IV a basal lamina component, which is bound by the dystrophin associated glycoprotein complex^23^. Compared to undamaged regions, the damaged regions at day three of either cohort appeared to be less uniform in terms of immunostaining signal intensity around the muscle fibre as well as in the thickness of the expression domain (Fig. 5M). The non-uniform and varying thickness pattern was evident in the leaky muscle area of *Atg16L1* muscle (Fig. 5M). This feature was resolved at day 6 (Fig. 5N).

## Discussion

Autophagy plays two major functions in animals. Firstly, its basal activity maintain the homeostatic function of tissues by acting as quality control mechanism, by promoting the degradation of malfunctioning organelles and toxic protein aggregates^3^. Additionally, autophagic activity can be induced in response a variety of stress conditions to act as an adaptive mechanism that underpins cell survival^24^. Skeletal muscle fibres rely heavily on autophagy due to their cellular nature. Muscle fibres are long lived cells and being unable to divide, they cannot dilute the accumulation of aberrant organelles and proteins through cell division^25^. Additionally autophagy mobilises the large stores of protein in skeletal muscle, which serve as source of amino acids to support the function of other organs during periods of stress^26,27^. It is now firmly established that too much or too little autophagic activity have catastrophic impact on muscle function and the health of mammals^12,13^. However, there is a dearth of knowledge regarding the physiological role of autophagy on skeletal muscle structure and its responsiveness to stress and injury. Here, we show that mild attenuation of autophagy reveals a role for this process in skeletal muscle homeostasis and ability to cope with acute nutritional and cellular stress.

Genetic diminution of autophagy by decreasing Atg16 function in mice did not impact on embryonic survival. Mice were born at expected Mendelian ratios and grew at the same rate as wild-type littermates (Fig. 1A and unpublished data). The skeletal muscle of *Atg16L1* mice appeared on the whole normal at the histological level. Skeletal muscle development seems to be refractory to decreases in the level of autophagy; the tissue forms differentiated fibres capable of contraction even when autophagy has been completely inhibited^28,29^. This contrasts to the situation in the brain, where neurogenesis has been shown to be affected even after mild perturbation in autophagy as in the *Atg16L1* mouse^10^. However, there are major differences in muscle when autophagy has been completely inhibited to when it is only mildly attenuated. The most striking and obvious difference is at the level of muscle weight. In this study we show that a decrease in autophagy manifests in a slight, albeit non-significant, decrease in muscle mass which contrasts starkly to the 40% decreases noted in muscle from mice where this activity was absent^28^. Nevertheless, we have been able to discern a physiological role for autophagy in muscle mass maintenance through the examination of fibre sub-types by showing that all fibres, categorised on MHC expression profile or oxidative capacity, rely on autophagy to attain their normal size. However, mild attenuation of autophagy had no role in the patterning of muscle fibres either on the basis of MHC profile or SDH expression. In agreement with the latter point, the capillary density of muscle fibres, a parameter regulated by the oxidative capacity of muscle fibres was not changed by a decrease in Atg16 function^30^. Molecular examination of skeletal muscle revealed that autophagy plays a major physiological role in preventing the build-up of ROS and limiting the harmful effects of this molecule. We show that even a mild attenuation of autophagy leads to at least a doubling of the level of ROS in muscle and significant increase in the degree of lipid peroxidation. One would predict, based on previous studies, that although the structure of muscle from *Atg16L1* mice appeared normal, that the build-up of ROS would have detrimental effects on muscle biology. Assessment of muscle function through the determination of specific force and contraction times were beyond the scope of this study but will be the focus of future investigations together with an ultra-structural profiling of *Atg16L1* muscle for signs of organelle damage.

The importance of autophagy in regulating the functioning of muscle was truly revealed when the *Atg16L1* mice were subjected to nutritional stress and acute damage. Here we found a significant response based on genotype to these insults. We show that attenuated Atg16 function led to significantly more muscle being lost in the first 12 h of starvation compared to wild-type mice. In both genotypes, starvation induced robust expression of *FoxO3a* a gene that encodes a master regulator of muscle atrophy through its ability to promote the expression of a number of L3 ubiquitin ligases including *Atrogin* and *Murf1*^31^. Given that these factors have been induced in both genotypes, it is important to offer an explanation of the greater degree of muscle loss in *Atg16L1* mice. Again, the level of protein synthesis controlled through AKT was similarly affected by starvation in both genotypes. However, there were key differences between the genotypes when we examined the impact starvation had on autophagy. In agreement with previous studies, autophagy, as measured by lipidated LC3 levels, was induced in the muscle of wild-type mice by starvation^32^. In contrast there was no induction of autophagy by starvation in *Atg16L1* mice. An important point to highlight is the presence of basal levels autophagy in fed *Atg16L1* mice, which was not enhanced by starvation. This contrasts other studies in which key regulators of autophagy have been completely removed. In these studies there was no basal, let alone inducible autophagy^28,29^. We believe that the blunted response in the *Atg16L1* genotype to starvation, in terms of autophagy, could be the key to increased levels of muscle loss identified in the first 12 h. We suggest that the absence of autophagy in the muscle of *Atg16L1* mice initiates other programmes to minimise stress and to deal with the build-up of abnormal proteins. Our data shows genotype-specific inhibition of eIF2α activity, a process usually controlled by the phosphorylation activity of PERK^33^. Inactivation of eIF2α not only inhibits protein synthesis as it is essential for the assembly of ribosomes but is also a component of the Unfolded Protein Response which becomes activated as a reaction to Endoplasmic Reticulum stress^34^. Furthermore, we show a significant increase in the degree of protein poly-ubiquitination only in the muscle of starved *Atg16L1* mice. We propose that in the absence of a starvation inducible autophagic response, the muscle of *Atg16L1* mice undergoes a period of accelerated muscle loss due to attenuated protein synthesis and high levels of poly-ubiquitin mediated polypeptide degradation. The latter two features have been documented in other stress related conditions, including age-related loss of mitochondrial function^35^. We note that by 24 h of starvation both genotypes had lost the same amount of muscle. Presently, we do not know why they rate of muscle loss in the *Atg16L1* mice slow in the second 12 h period following starvation. However recent work has shown that mechanisms exist that prevent excessive muscle loss in the face of degeneration and atrophy and are controlled by the expression of Myostatin^36^. Herein it is thought that muscle loss can be mediated by Myostatin expression, but that it is down-regulated if it becomes excessive. In such a scenario, one would predict that in the first 12 h of starvation, the expression of Myostatin would be higher in *Atg16L1* mice than WT. Thereafter, due to excessive muscle loss, its expression in *Atg16L1* mice would be decreased in comparison to WT. However we believe that muscle loss in the WT cohort is coordinated where as in the *Atg16L1* mice it is not. Future work will investigate the hypothesis that although the two cohorts had experienced the same degree of muscle loss by 24 h that deregulated atrophy in the absence of an inducible autophagic programme would have negatively impacted on the physiology of *Atg16L1* tissue and through the assessment of Myostatin, Activin and GDF11 levels in the circulation.

Lastly, we show that inducible autophagy has a major impact on muscle regeneration. Our results show that autophagy has a role to play in the regeneration of new fibres, the clearance on dying fibres and the stability of muscle fibres to CTX insult. Our work shows that at day 6 after CTX injury the size of newly formed muscle fibres of *Atg16L1* were about half the size of wild-types. We propose that the deficit in the size of newly formed muscle fibres could in part be due to the impact of attenuated autophagy on the activity of Satellite Cells (SC) the resident stem cells of skeletal muscle^37,38^. Satellite cells normally exist in a quiescent state with low metabolic activity^39^. They contain granules composed of translation initiation factors, RNA binding proteins and mRNA and are poised for activation and proliferation, a process kept in check through the inhibition of protein synthesis, mediated to a large extent by the phosphorylation of eIF2α^40,41^. Autophagy is required for satellite cells to come out of their quiescent state through its ability to generate ATP needed for activation and proliferation^39^. Furthermore in the context of muscle regeneration, autophagy may also control the differentiation programme as it has been recently been shown that it promotes the Notch degradation, a process that would promote the development of committed myoblasts at the expense of stem cells^10^. We show that regenerating muscle from *Atg16L1* mice had a greater proportion of muscle stem cells (Pax7^+^/MyoD^−^) and lower proportion of muscle precursors (Pax7^+^/MyoD^+^) or committed myoblasts (Pax7^−^/MyoD^+^) than wild-type. Therefore, we postulate that the attenuated muscle regeneration in the *Atg16L1* mice, evidenced by the smaller size of newly formed fibres (eMHC^+^) could be due to a lack of activation of stem cells after damage. This may lead to fewer myoblasts being generated to drive regeneration at its normal pace. These results are concordant for a role for autophagy in the differentiation of stem cells which has been previously described in the brain, gut and bone marrow^10^.

Our work also identified a novel role for autophagy in the maintenance of sarcolemma integrity. We showed that injection of CTX in the TA muscle of *Atg16L1* mice resulted in the fibres becoming leaky and infiltrated by circulating Ig molecules. Although these fibres exhibited additional molecular abnormalities such as the intra-fibre localisation of dystrophin and nNOS, they did not die and were able to essentially restore the two proteins to their normal site (albeit to a lower level than normal for nNOS). CTX brings about muscle fibre death through the activity of Phospholipase A2 (PLA_2_) which binds to proteins on the sarcolemma followed by hydrolysis of phospholipids^42,43^. Puncturing of the sarcolemma by PLA_2_ causes a rapid fall in the resting membrane potential and a rise in the cytosolic concentration of Ca^2+^ which activates not only proteases but also leads to the demise of mitochondria and ultimately fibre death^44,45^. The mechanism by which autophagy makes muscles fibres resilient to the action CTX needs to be determined but one important feature discovered by this work is that leaky fibres with altered sub-sarcolemma organisation (intra-fibre dystrophin and nNOS) are not necessarily destined to die. It is generally accepted that elevated levels of cytosolic Ca^2+^ are key mediators of muscle fibre death in muscular dystrophies or by the action of snake venoms. However the muscle fibre has a capacity to buffer against changes in cytosolic Ca^2+^ for example through the activity of mitochondria^46^. We propose that at sites of high CTX concentration, the cytosolic levels of Ca^2+^ cannot be buffered to prevent the activation of fibre necrosis, resulting in not only the death of the fibre but also infiltration by macrophages and the activation of satellite cells to regenerate lost tissue. However, fibres that experience a lower level of CTX become leaky but are able to buffer the rise in Ca^2+^ to levels insufficient to activate necrosis and all the other associated cellular events. Herein we speculate that autophagy is required to maintain membrane integrity and in its absence fibres become leaky at low levels of CTX which nevertheless are able to keep Ca^2+^ to sub-lethal levels.

In summary, our study shows that modest alteration in autophagy reveals a role for this process in muscle fibre growth as well as coping with starvation and acute injury. Importantly this work demonstrated the value on hypomorphic models of autophagy, where this process is altered slightly which may expose the physiological role played by this process in animals.

## Methods

### Mice

Healthy BL6/BC122 WT or ATG16L1 mice were bred and maintained in accordance to the Animals (Scientific Procedures) Act 1986 (UK) and approved by the University of Reading and University of Cambridge local ethics committees. The experiments were performed under a project license from the United Kingdom Home Office in agreement with the Animals (Scientific Procedures) Act 1986 (PPL70/7516). The University of Reading Animal Care and Ethical Review Committee (AWERB) approved all procedures. Animals were humanely sacrificed via Schedule 1 killing between 8:00–13:00. Mice were housed under standard environmental conditions (20–22 °C, 12–12 hr light–dark cycle) and provided food and water ad libitum. 8-week-old mice were used in this study.

### Starvation protocol

Mice were moved from communal housing in normal cages to single housing cages containing a water source as well as copious inedible bedding material to mitigate any effects of hypothermia. Animals were monitored every 6 hours and maintained under starvation conditions for a maximum of 24 h.

### Immunohistochemistry

Dissected and frozen muscles (EDL, Soleus and TA) were mounted in Tissue Tech Freezing Medium (Jung) cooled by a dry ice/ethanol bath and cryosectioned at 10 µm thick sections using a Bright OTF cryostat (OTF/AS-001/HS) and placed onto glass slides for immunohistochemistry. Slides were removed from −80 °C, air dried for 30 minutes at room temperature (RT) prior to three washes in 1 × PBS. Muscle sections were incubated in permeabilisation buffer solution (0.952 g Hepes, 0.260 g MgCl2, 0.584 g NaCl, 0.1 g Sodium azide, 20.54 g Sucrose and 1 ml Triton X-100 in 200 ml dH2O) for 15 minutes at room temperature. To remove excess permeabilisation buffer, another three five minutes washes in 1 × PBS were performed before the application of block wash buffer (PBS with 5% foetal calf serum (v/v), 0.05% Triton X-100) for 30 minutes at room temperature.

Primary antibodies were pre-blocked in wash buffer for 30 minutes prior to application onto muscle sections overnight at 4 °C. In order to remove the primary antibodies, muscle sections were washed three times in wash buffer with each wash lasting 10 minutes. Primary antibodies were identified using Alexa flour 488, 594 and 633 secondary antibodies. All secondary antibodies were pre-blocked in wash buffer for minimum of 30 minutes prior to their application onto the slides (1 hr in the dark at room temperature). Thereafter the muscle sections were washed three-10 minutes washes in PBS to remove the secondary antibody. Finally, slides were mounted in fluorescent mounting medium, and myonuclei were visualized using (2.5 μg/ml) 4,6-diamidino-2-phenylindole (DAPI). Details of primary and secondary antibodies are given in Supplementary Data File 1.

### Succinate dehydrogenase (SDH) staining

Muscle sections were incubated for 3–5 minutes at room temperature in a sodium phosphate buffer containing 75 mM sodium succinate (Sigma), 1.1 mM Nitroblue Tetrazolium (Sigma) and 1.03 mM Phenazine Methosulphate (Sigma). Samples were then fixed in 10% formal-calcium and cleared in xylene prior to mounting with DPX mounting medium (Fisher). Densitometry of the samples was performed on a Zeiss Axioskop2 microscope mounted with an Axiocam HRc camera. Axiovision Rel. 4.8 software was used to capture the images.

### Dihydroethidium (DHE) and 4-hydroxy-2-nonenal (4-HNE) staining

Muscle sections were rehydrated in 1 × PBS for 5 minutes before being incubated with 10 µM of DHE (Sigma-Aldrich 7008) 30 minutes at 37 °C or 4-HNE antibody. The slides were then washed in 1 × PBS three times with each wash lasting 5 minutes. Finally, slides were mounted in fluorescent mounting medium, and myonuclei were visualised using (2.5 µg/ml) 4,6-diamidino-2-phenylindole (DAPI).

### Quantitative PCR

Frozen Gastrocnemius muscles were cut into three thirds, and (40–50 mg) pieces of each muscle pulverised and solubilised in TRIzol® (Fisher) using a tissue homogenizer (QIAGEN). Total RNA was isolated and purified using the RNesay Mini Kit (Quigen, Manchester, UK, 74104). RNA concentrations were measured using the Nanodrop 2000 (Thermo Scientific). RNA (5 μg) was reverse-transcribed to cDNA with SuperScript II Reverse Transcriptse (Invitrogen) and analysed by quantitative real-time RT-PCR on a StepOne Plus cycler, using the Applied Biosystems SYBR-Green PCR Master Mix. Primers were designed using the software Primer Express 3.0 (Applied Biosystems). Relative expression was calculated using the ΔΔCt method with normalization to the housekeeping genes *cyclophilin-B* and *hypoxanthine-guanine phosphoribosyltransferase (HPRT*). Specific primer sequences are given in Supplementary Data File.

### Capillary density quantification

Muscles sections (EDL and Soleus) were immunostained using CD31 antibody in order to identify the number of blood vessels that serve each muscle fibre. Quantification of capillary density was performed by selecting central area of muscle sections that includes approximately 150 to 200 fibres The number of muscle fibres were determined as well as the number of CD31^+^ in the selected area. Thereafter the ratio of blood capillaries to fibres (C:F) was calculated.

### Antigen retrieval immunostaining for Pax7/MyoD quantification

In order to quantify positive Pax7 and MyoD cells and to improve the presentation of our target antigens and enhance immunoreactivity, pre-treatment with the antigen retrieval reagents was performed. Sectioned slides were fixed in 4% PFA for 20 minutes at RT and incubated in 100% methanol for 5 minutes before being immersed into the preheated retrieval solution for 2–10 minutes. Following the incubation, three five minutes washes in 1 × PBS were performed to remove excess retrieval solution from the slides. Sectioned slides were then pre-blocked in wash buffer for 30 minutes at room temperature before being incubated with primary antibodies (Pax7 and MyoD) overnight at 4 °C. Primary antibodies were identified using anti- mouse or anti rabbit Alexa fluor 488, 594 and 633 secondary antibodies. Steps of day 2 of immunostaining protocol were followed before slides being mounted in fluorescent mounting medium and coverslip was placed over the top.

Quantification of positive Pax7 and MyoD cells was performed by counting nuclei in the damaged areas of TA muscles, then counting the number of Pax7^+^ expressing cells or MyoD^+^ expressing cells or cells that expressed bothPax7^+^/MyoD^+^. Example of Pax7/MyoD stating is given in Supplementary figure X. A positive signal was only noted where the expression of either epitope co-localised to a DAPI stained nuclei. Data is presented as the number of events per square millimetre of damaged muscle. Example of Pax7 and MyoD immunohistochemistry of damaged muscle is given in Supplementary Figure 2.

### Western blotting

Frozen gastrocnemius muscles were powdered and lysed in a buffer containing 4 M Urea, 125 mM Tris pH 6.8, 4% SDS and protease inhibitor cocktail set 1 (calbiochem). Protein concentration was determined via Bradford Assay (BioRad). 30 µg protein for each sample was resolved on a 4–12% SDS page gel (Life Technologies) prior to 3 hour transfer at 30 V on to a PVDF membrane (Thermo Scientific) for immunoblotting (details of antibodies used are given in supplementary information file). Blots were visualised with ECL™ Prime Western Blotting System (GE Healthcare). Images of Western blots were taken using ImageQuant LAS 4000 mini, ensuring they were below signal saturation. Protein expression was quantified using Fiji software and normalised to the expression of housekeeping proteins.We followed the journals guidelines for the presentation of Western blot data. All antibodies were initially characterised so that blotting conditions gave unequivocal recognition of the correct epitope, wherein entire blotting membrane was used. Examples of such blots are given in Supplementary Figure 3. Thereafter after blotting, membranes were sometimes cuts (guided by the molecular weight of a particular epitope and processed with different antibodies. This practice maximised the data return for a given amount of biological sample. Presentation of individual bands sometimes required cropping for purely repositioning purposes and was used for clarity and presentational purposes alone.

### Semi-quantitative Measures of Sarcolemma Protein Expression

Intensity of signals from the protein of interest was measured as previously described^47,48^. Briefly, membrane signal intensities of approximately 30 muscle fibres of each MHC phenotype (IIA and IIB) in all muscle sections from wild type and *Atg16L1* were analyzed. Images of muscle sections after immunostaining were firstly captured using Zeiss AxioImager A1, then tiles assembled into whole muscle image using Photoshop CS3 as JPEG files. Fiji software was used to measure the signal from the JPEG pictures of the area of interest after images had been corrected for background to avoid signal saturation. To calculate relative signal intensity levels, individual measurements from treated and control fibres were taken as a percentage of mean of control samples. The same process was used for DHE and 4-HNE quantification.

### Imaging and analysis

Zeiss AxioImager A1 microscope was used to examine immunofluorescent stained sections, and images were captured using an Axiocam digital camera with Zeiss Axiovision computer software version 4.8.

### Statistical Analysis

Data are presented as mean ± SE. Significant differences between two groups were performed by two-tailed Student’s t test for independent variables. Differences among groups were analyzed by two-way ANOVA followed by Bonferroni multiple comparison tests as appropriate. Statistical analysis was performed on GraphPad Prism software. Differences were considered statistically significant at *p < 0.05, **p < 0.01, or ***p < 0.001.

### Data availability

All relevant data related to this manuscript are available from the authors upon reasonable request.

## Electronic supplementary material

Supplementary information and figures
