# Author Correction: Attenuation of autophagy impacts on muscle fibre development, starvation induced stress and fibre regeneration following acute injury

**DOI:** 10.1038/s41598-021-04140-8

**Published:** 2021-12-29

**Authors:** Andrea Paolini, Saleh Omairi, Robert Mitchell, Danielle Vaughan, Antonios Matsakas, Sakthivel Vaiyapuri, Thomas Ricketts, David C. Rubinsztein, Ketan Patel

**Affiliations:** 1 School of Biological Sciences, University of Reading, Reading, UK; 2 Molecular Physiology Laboratory, Centre for Atherothrombotic & Metabolic Disease, Hull York Medical School, Hull, UK; 3 School of Pharmacy, University of Reading, Reading, UK; 4 Cambridge Institute for Medical Research, Department of Medical Genetics, University of Cambridge, Cambridge, UK; 5 UK Dementia Research Institute, Cambridge Biomedical Campus, Cambridge, UK

Correction to: *Scientific Reports* https://doi.org/10.1038/s41598-018–27429-7, published online 13 June 2018

The original version of this Article contained errors.

In Figure 5, the distance that “leaky” images were taken from the damaged tissue was not consistent, and there was a partial overlap of the “leaky” and undamaged images for Figure 5D and 5J. In addition, for some panels, the images presented were from different muscle sections.

**Figure 5:**
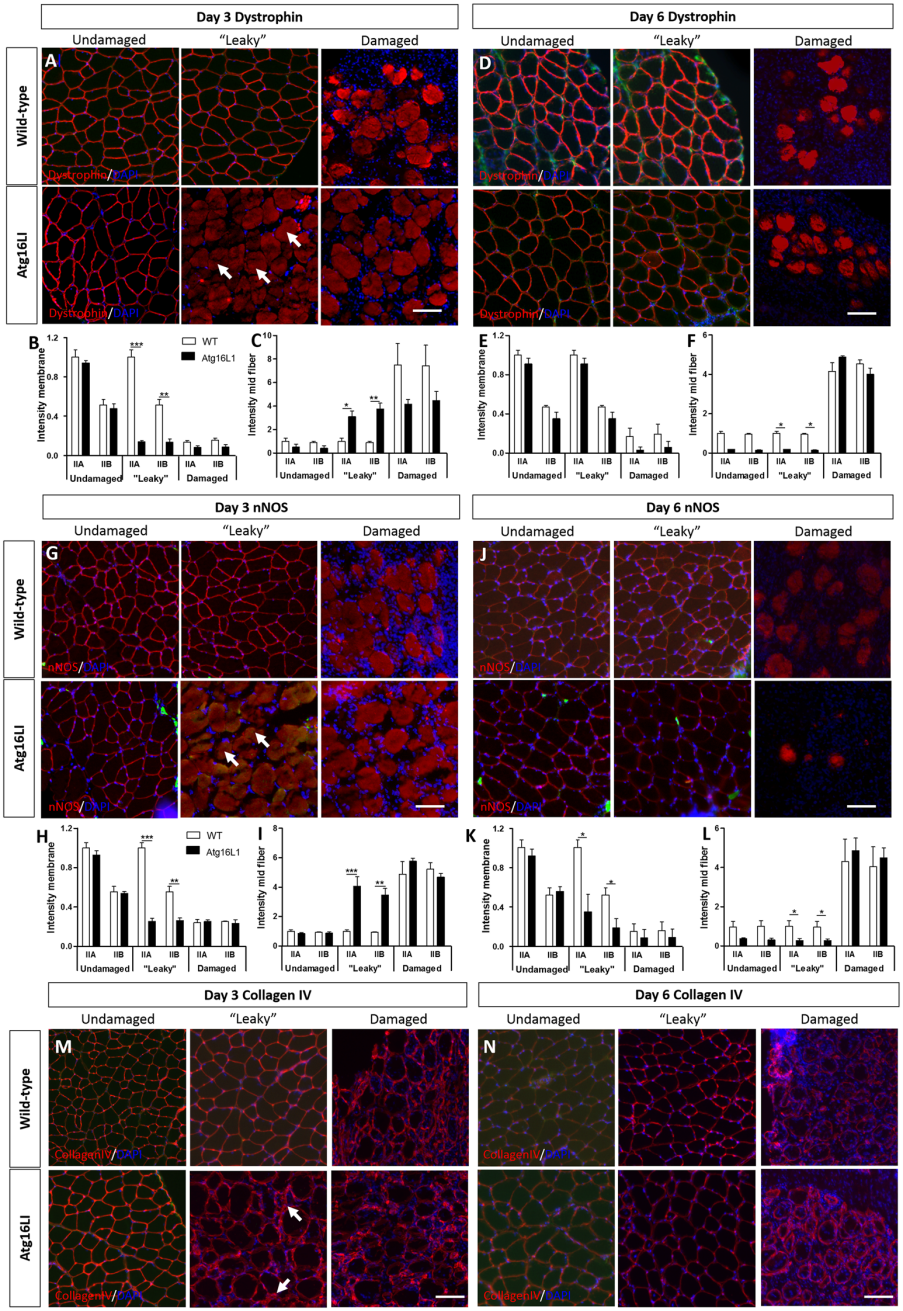
The impact of attenuated autophagy following acute muscle damage on components of the dystrophin associated glycoprotein complex. (**A**) Immunocytochemical analysis of dystrophin expression in undamaged, leaky and damaged regions at day three. Arrows show presence of dystrophin within the fibre in leaky area in Agt16L1 muscle. Quantification of dystrophin levels at the sarcolemma (**B**) and with the fibre (**C**) with relation to muscle fibre type in three regions of interest at day three. Note all quantification levels compared to a baseline of 1 of undamaged type IIA fibres. (**D**) Immunocytochemical analysis of dystrophin expression in undamaged, leaky and damaged regions at day six. Quantification of dystrophin levels at the sarcolemma (**E**) and with the fibre (**F**) with relation to muscle fibre type in three regions of interest at day six. (**G**) Immunocytochemical analysis of nNOS expression in undamaged, leaky and damaged regions at day three. Arrows highlight nNOS within the fibre in leaky area in Agt16L1 muscle. Quantification of nNOS levels at the sarcolemma (**H**) and with the fibre **(I**) with relation to muscle fibre type in three regions of interest at day three. (**J**) Immunocytochemical analysis of nNOS expression in undamaged, leaky and damaged regions at day six. Quantification of nNOS levels at the sarcolemma (**K**) and with the fibre (**L**) with relation to muscle fibre type in three regions of interest at day six. Immunocytochemical analysis of Collagen IV expression at day three (**M**) and day six (**N**) in undamaged, leaky and damaged regions in the two cohorts. Arrows in (**M**) highlight non-uniform collagen expression in Atg16L1 muscle at day three. Scale bar 100 µm. n = 3/4 8-week-old-male for each cohort. *p < 0.05, **p < 0.01 and ***p < 0.001. Statistical analysis between two groups performed by two-tailed Student’s t test for independent variables.

The original Figure 5 and accompanying legend appears below.

The original Article has been corrected.

