# Supplementary material for: Attenuation of autophagy impacts on muscle fibre development, starvation induced stress and fibre regeneration following acute injury
Source: Sci Rep. 2018 Jun 13;8:9062. doi: 10.1038/s41598-018-27429-7 (PMC5998118; doi:10.1038/s41598-018-27429-7)

**Supplementary Data File**

**Title:**

**Attenuation of autophagy impacts on muscle fibre development, starvation induced stress and fibre regeneration following acute injury**

**Authors**

**Andrea Paolini^1,^*, Saleh Omairi^1,*^, Robert Mitchell^1^, Danielle Vaughan^1^, Antonios Matsakas^2^, Sakthi Vaiyapuri^3^, Thomas Ricketts^4^, David C. Rubinsztein^4,5^, Ketan Patel^1,6^**

**Afflation**

1. School of Biological Sciences, University of Reading. Reading. UK
2. Molecular Physiology Laboratory, Centre for Atherothrombotic & Metabolic Disease, Hull York Medical School, Hull, UK
3. School of Pharmacy, University of Reading. Reading. UK
4. Cambridge Institute for Medical Research, Department of Medical Genetics, University of Cambridge, UK
5. UK Dementia Research Institute, Cambridge Biomedical Campus, Cambridge, UK
6. Corresponding author School of Biological Sciences, University of Reading, Whiteknights, Reading, RG6 6UB. Email: [ketan.patel@reading.acc.uk](mailto:ketan.patel@reading.acc.uk)

*Equal contribution

**Primary Antibodies:**

| **Antigen** | **Type** | **Immunoglobulin** | **Species** | **Dilution** | **Supplier** |
| --- | --- | --- | --- | --- | --- |
| **MYHCI** | Monoclonal | IgG | Mouse | 1:1 | DSHB A4.840 |
| **MYHCIIA** | Monoclonal | IgG | Mouse | 1:1 | DSHB A4.74 |
| **MYHCIIB** | Monoclonal | IgM | Mouse | 1:1 | DSHB BF.F3 |
| **CD31** | Monoclonal | IgG | Rat | 1:150 | AbD serotec MCA2388 |
| **Collagen type IV** | Polyclonal | IgG | Rabbit | 1:500 | Abcam, 6586 |
| **Dystrophin** | Polyclonal | IgG | Rabbit | 1:200 | Abcam, 15277 |
| **nNOS** | Polyclonal | IgG | Rabbit | 1:200 | Santa Cruz Bio,648 |
| **MYH3** | Monoclonal | IgG | Mouse | 1:200 | Santa Cruz Biot, sc-53091 |
| **4-HNE** | Polyclonal | IgG | Rabbit | 1:200 | Abcam, 46545 |
| **Pax7** | Monoclonal | IgG | Mouse | 1:1 | DSHB |
| **MyoD** | Polyclonal | IgG | Rabbit | 1:200 | Santa Cruz Biot, # sc-760 |

**Secondary Antibodies:**

| **Antibody** | **Species** | **Dilution factor** | **Supplier** |
| --- | --- | --- | --- |
| **Alexa fluor 633 anti‐mouse** | Goat | 1:200 | Life Technologies # A20146 |
| **Alexa fluor 488 anti‐mouse** | Goat | 1:200 | Life Technologies # A11029 |
| **Alexa fluor 488 anti‐rabbit** | Goat | 1:200 | Life Technologies # A11034 |
| **Alexa fluor 594 anti‐rabbit** | Goat | 1:200 | Life Technologies # A11037 |

**Western blotting antibodies**

**Primary Antibodies:**

| **Antigen** | **Species** | **Dilution** | **Supplier** |
| --- | --- | --- | --- |
| **pFOXO3a (Ser320)** | rabbit | 1:1000 | Bioss#bs-3138R |
| **FOXO3a** | rabbit | 1:1000 | Cell Signalling #2497 |
| **pAKT (Ser473)** | rabbit | 1:2000 | Cell Signalling #4060 |
| **AKT** | rabbit | 1:1000 | Cell Signalling #9272 |
| **pS6 (Ser 240/244)** | rabbit | 1:1000 | Cell Signalling #2215 |
| **S6** | rabbit | 1:1000 | Cell Signalling # 2217 |
| **pEIF2a (phospho S51)** | rabbit | 1:1000 | Abcam, ab32157 |
| **EIF2a** | rabbit | 1:1000 | Cell Signalling #9722 |
| **OPA1** | mouse | 1:1000 | BD Biosciences, 612607 |
| **P62** | rabbit | 1:1000 | Sigma, P0067 |
| **LC3** | rabbit | 1:1000 | Cell Signalling #2775 |
| **EP8589** | rabbit | 1:1000 | Abcam, ab140601 |
| **GAPDH** | mouse | 1:5000 | EMD Millipore, 2842113 |

**Secondary antibodies:**

| **Antibody** | **Dilution factor** | **Supplier** |
| --- | --- | --- |
| **Goat anti rabbit (IgG) HRP** | 1:5000 | Thermofisher, 65-6120 |
| **Rabbit anti mouse HRP** | 1:5000 | DAKO, P0260 |

**qPCR primers Sequence**

| **mCYCLO.F** | **TGG AGA GCA CCA AGA CAG ACA** |
| --- | --- |
| **mCYCLO.R** | **TGC CGG AGT CGA CAA TGA T** |
| **mHPRT.F** | **GCTCGAGATGTCATGAAGGAGAT** |
| **mHPRT.R** | **AAAGAACTTATAGCCCCCCTTGA** |
| **mFoxO.F** | **GCTGGGTGTCAGGCTAAGAG** |
| **mFoxO.R** | **AGGGGTGAAGGGCATCT** |
| **mMuRF1.F** | **ACCTGCTGGTGGAAAACATC** |
| **mMuRF1.R** | **CTTCGTGTTCCTTGCACATC** |
| **mAtrogin-1F** | **GCAAACACTGCCACATTCTCTC** |
| **mAtrogin-1R** | **CTTGAGGGGAAAGTGAGACG** |

**Supplementary data Figure 1.** Effect of starvation on muscle fibres of the soleus. (A) Morphometric analysis of soleus fibres after 12 h of starvation and (B) at 24h after food withdrawal.


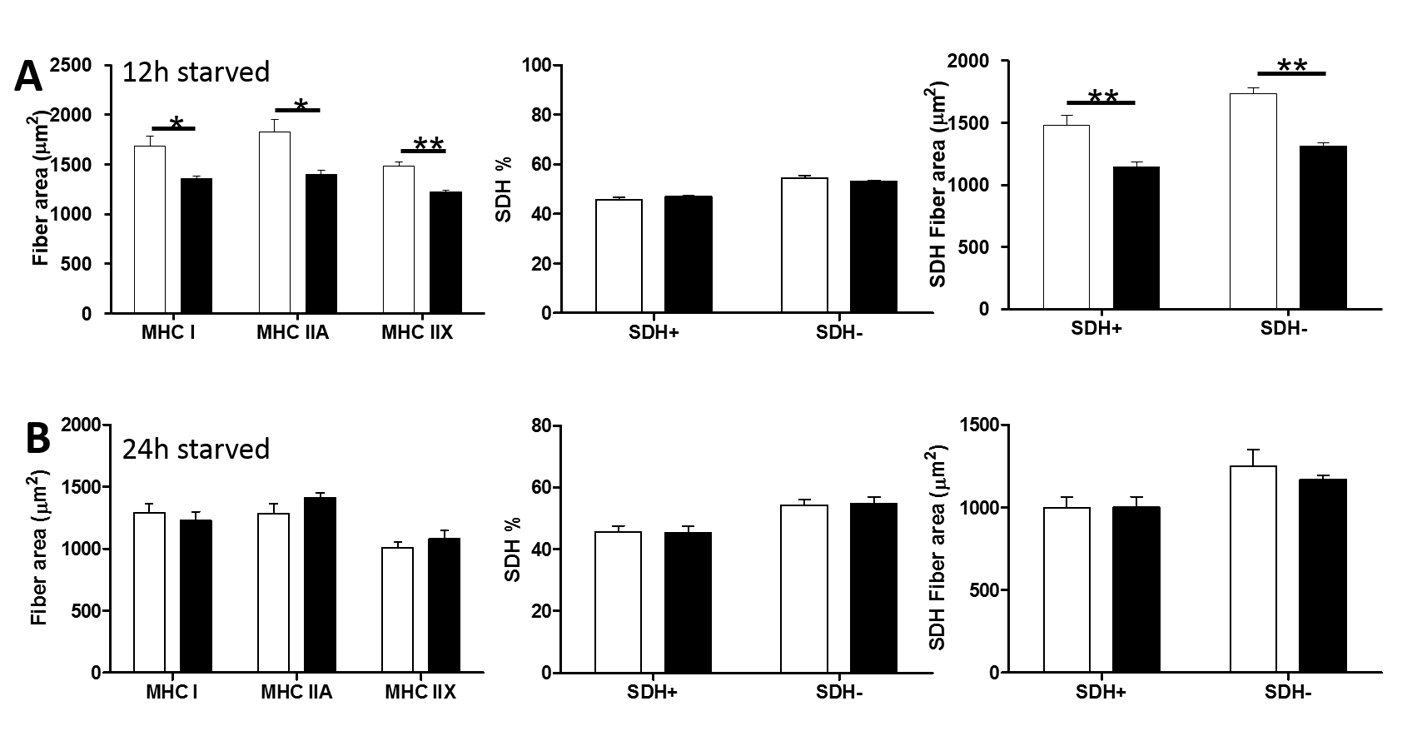


**Supplementary data Figure 2.** Image of immunohistochemical staining for expression of Pax7 and MyoD of Atg16L1 TA muscle 6 days after injury with cardiotoxin. Green arrows indicate expression of nuclear PAx7, red arrows indicate expression of MyoD in the nuclei and yellow arrows indicate nuclei expressing both Pax7 and MyoD.


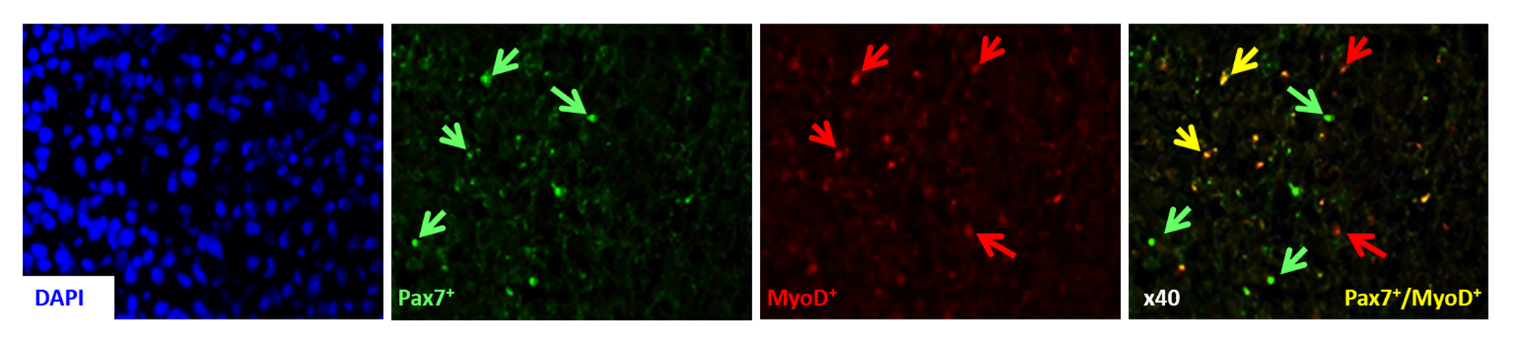


**Supplementary data Figure 3.** Examples of whole membranes examined for the abundance of FoxO3a, S6 and LC3 in muscle extracts by Western Blotting.


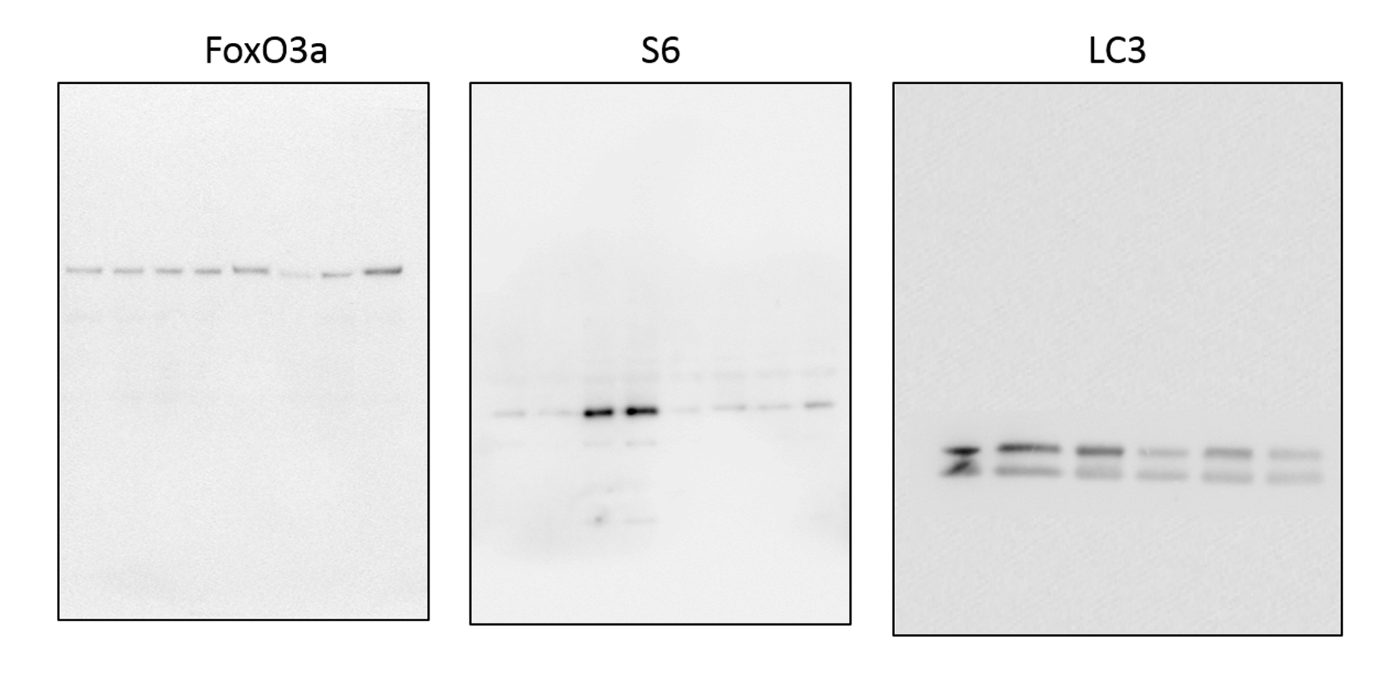

Supplement: Supplementary file 1 — Supplementary information and figures [file 41598_2018_27429_MOESM1_ESM.docx]
